# Anti-Thrombotic Therapy Following Transcatheter Structural Heart Intervention

**DOI:** 10.3390/jcm15083175

**Published:** 2026-04-21

**Authors:** Francesco Tartaglia, Giulia Antonelli, Alessandro Gabrielli, Mauro Gitto, Arif A. Khokhar, Francesca Soriente, Pier Pasquale Leone, Damiano Regazzoli, Ole de Backer, Antonio Mangieri, Giulio Stefanini

**Affiliations:** 1 Department of Biomedical Sciences, Humanitas University, Pieve Emanuele, 20072 Milan, Italy; francesco.tartaglia@humanitas.it (F.T.);; 2 IRCCS Humanitas Research Hospital, Rozzano, 20089 Milan, Italy; 3 The Heart Center, Rigshospitalet, Copenhagen University Hospital, 2100 Copenhagen, Denmark; 4 Montefiore Einstein Center for Heart and Vascular Care, Montefiore Medical Center, Albert Einstein College of Medicine, Bronx, NY 10461, USA

**Keywords:** antithrombotic, TAVI, LAAO, bleeding

## Abstract

Transcatheter structural heart interventions, including aortic, mitral and tricuspid valve replacement or repair, and patent foramen ovale, atrial septal defect, and left atrial appendage closure, have dramatically expanded over the past two decades, providing substantial improvements in both clinical outcomes and quality of life. These interventions are performed in a high-risk patient population, which is at risk for both thrombotic and bleeding complications. The introduction of prosthetic devices into the arterial or venous circulation under heterogeneous hemodynamic conditions inevitably increases the risk for thrombotic events and thromboembolic complications. Consequently, the selection of antithrombotic therapy (AT) regimen and its duration is complex and should be tailored to each patient’s risk profile, balancing the expected risk and benefits. This state-of-the-art review critically examines the thrombotic risks inherent to transcatheter structural heart interventions, synthesizes available evidence and current guidelines recommendations on antithrombotic management, and defines persisting gaps in knowledge while discussing the most relevant ongoing clinical trials.

## 1. Introduction

**Figure 1:**
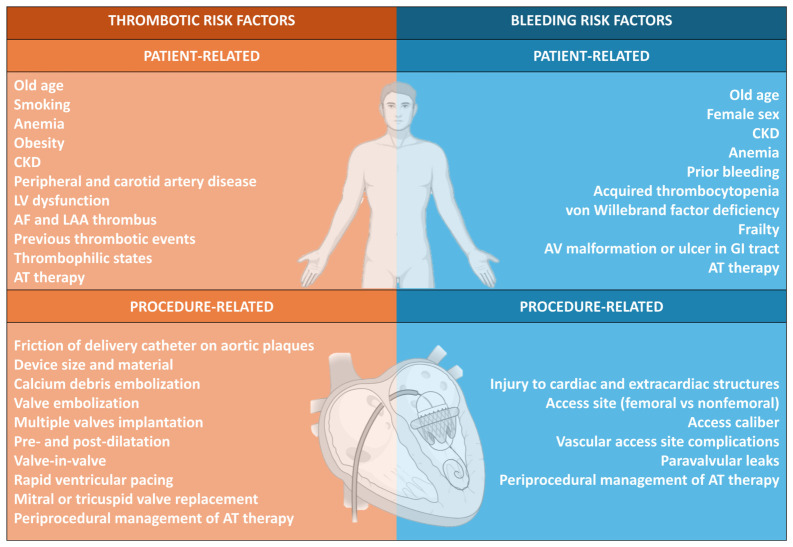
Thrombotic and bleeding risk factors in patients undergoing transcatheter structural heart interventions. Abbreviations: AF: atrial fibrillation; AT: antithrombotic; AV: arteriovenous; CKD: chronic kidney disease; GI: gastrointestinal; LAA: left atrial appendage; LV: left ventricle.

The rapid expansion of transcatheter structural heart interventions has profoundly reshaped the management of valvular and non-valvular heart disease [1,2]. Exposure of cardiac devices to the bloodstream requires post-procedural antithrombotic therapy (AT) to prevent device thrombosis and its possible complications. However, the optimal pharmacological strategy depends on a combination of patient-related and device-related factors, and the prevention of thromboembolic events must be carefully balanced against the risk of bleeding in an often elderly and comorbid population [3] (Figure 1).

As indications continue to expand and procedural volumes increase, AT has emerged as a critical determinant of clinical outcomes, yet it is still the subject of ongoing investigations. In this review, we aim to provide a contemporary overview of the available evidence and explore unresolved clinical questions in the field of AT after transcatheter structural heart interventions.

## 2. Transcatheter Aortic Valve Implantation

### 2.1. Thrombotic Risk

Transcatheter aortic valve implantation (TAVI) is currently recommended as the treatment of choice for patients older than 70 years with severe, symptomatic aortic stenosis (AS) across all surgical risk categories [1,2]. Thrombotic complications may arise in either the peri- or post-procedural phase. Peri-procedural stroke occurs in ~3% of procedures and is linked to embolization from the aorta and the aortic valve, as well as to hypotensive states during the procedure, with events typically clustered within the first week after the procedure [4]. Late-occurring strokes are related to the patient’s risk profile rather than to the TAVI procedure itself. In contrast, thromboembolic myocardial infarction is rare (~1%) and should be differentiated from non-thrombotic causes of coronary obstruction [5]. Late coronary obstruction from thrombus formation around the prosthesis has also been reported [6].

**Figure 2:**
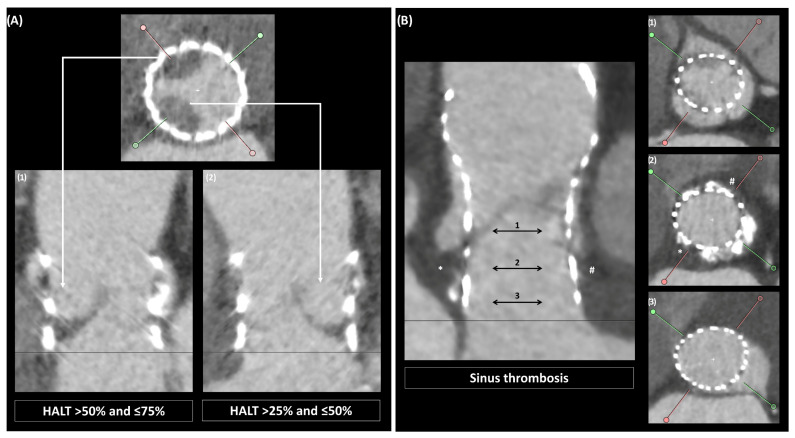
Subclinical leaflet thrombosis after TAVI. (**A**) shows a case of HALT involving 2 leaflets of a short frame THV (SAPIEN 3, Edwards Lifesciences, Irvine, CA, USA). According to VARC-3 definitions, HALT would be classified as “>50% and ≤75%” for leaflet 1, and “>25% and ≤50%” for leaflet 2. (**B**) shows a case of native sinus thrombosis, mainly involving the non-coronary (*) and the right sinuses (#). Abbreviations: HALT: hypoattenuated leaflet thickening; TAVI: transcatheter aortic valve implantation; THV: transcatheter heart valve.

A thrombus can also affect the implanted prosthetic valve itself. Clinically significant valve thrombosis, defined as clinical sequelae of a thromboembolic event or worsening valve function in the presence of relevant hemodynamic valve deterioration or confirmatory imaging [7], is a rare event (<1%) [8] that should be treated with anticoagulation, thrombolysis or reintervention, according to the clinical scenario [1,2] (Table 1). On the other hand, subclinical leaflet thrombosis (SLT) consists of hypoattenuated leaflet thickening [HALT], which may be associated with restricted leaflet motion [RLM] (Figure 2). SLT has been reported in 6–30% of contemporary bioprostheses [9,10,11], with a higher incidence in intra-annular devices [12]. Recently, the presence of perivalvular thrombosis has been described (including subvalvular and sinus thrombosis), with an incidence that seems to be even higher than HALT [10,13]. Although some studies have shown an association between HALT and neurological events, the relationship still remains debated [14,15]. The low incidence of embolic events may make it difficult to detect an association between HALT and clinical outcomes. Similarly, the link between HALT and clinically relevant hemodynamic deterioration is unclear, and further studies are needed to determine whether preventing or treating HALT leads to clinical benefit. Therefore, no strong recommendation can be made regarding the relevance of routine CT screening after TAVI. Until more evidence becomes available, imaging should remain symptom-driven.

HALT has also been associated with valve underexpansion and flow distortion. Underexpansion may lead to leaflet deformation, pinwheeling and thrombosis [16,17]. Consequently, implant optimization strategies, such as routine postdilation, may reduce the incidence of HALT; however, further data are needed to confirm this hypothesis. Similarly, flow distortion has been proposed as a potential risk factor for thrombosis [18]. Novel device designs to promote laminar rather than turbulent flow across the prosthesis are under investigation [19].

If increased transvalvular gradients are associated with computed tomography (CT)-confirmed RLM and/or HALT, then anticoagulation therapy is recommended [1,2] (Table 1). This is based on three RCTs, which have consistently demonstrated that anticoagulation is associated with a reduced incidence of HALT, compared to antiplatelet therapy alone, as discussed below [20,21,22] (Table 2).

Multiple factors contribute to thrombus formation on the bioprostheses, including flow distortion [18] and the presence of the stent frame and leaflets, whose material differs across devices (Table 3). Since endothelization is not uniform on all stent struts [32], the persistent exposure of prothrombotic material demands a long-term AT strategy. This inevitably increases the bleeding risk in this already fragile population, with rates of major and life-threatening bleeding varying between 2% and 20% [26,27,30,31], although these rates are currently decreasing thanks to procedural refinements, inclusion of lower-risk patients, and better AT selection [33].

### 2.2. Current Recommendations

Despite the yearly increase in procedural volume [34], data concerning post-TAVI antithrombotic strategies are relatively limited, especially compared to the high number of randomized controlled trials (RCTs) evaluating AT in the setting of post-percutaneous coronary intervention (PCI).

Both European and American guidelines recommend single antiplatelet therapy (SAPT) with aspirin for at least 12 months in patients with no previous indication for anticoagulation, and oral anticoagulation (OAC) alone in those with a previous indication [1,2] (Table 1). The American guidelines still include a class IIb indication for dual antiplatelet therapy (DAPT) and vitamin-K antagonists (VKA) in post-TAVI patients, but these indications appear outdated and are likely to be revised in the next edition of the guidelines. No specific AT regimen is suggested for patients who have undergone PCI. A European consensus suggests reducing DAPT to a maximum of 6 months in patients undergoing TAVI who have undergone recent PCI (<3 months), as TAVI patients are considered at higher risk of bleeding (mostly due to age and comorbidities) [35].

Available evidence is described in Table 2 and briefly discussed as follows.

### 2.3. Patients Without Previous Indication for Anticoagulation

DAPT with aspirin plus clopidogrel (or ticlopidine), which was the standard in the early TAVI era [36], was tested against aspirin alone by four RCTs with progressively larger sample sizes [23,24,25,26]. In all of them, no differences in ischemic outcomes were found between the DAPT and SAPT cohorts. However, DAPT was associated with a higher rate of vascular complications in the SAT-TAVI (single antiplatelet therapy for TAVR) trial [24] and with a threefold higher rate of major or life-threatening bleeding in the ARTE (Aspirin Versus Aspirin and Clopidogrel Following TAVR) trial [25]. The largest study to compare SAPT and DAPT was the POPular TAVI (Antiplatelet Therapy for Patients Undergoing TAVR) trial (cohort A) [26], which found that bleeding events were almost doubled in the DAPT arm, of which vascular access-related bleeding was the most common.

On this basis, the use of DAPT after TAVI is now limited to concomitant extra-TAVI indications, primarily including PCI or peripheral interventions related to the procedure. An observational study reported a trend towards better outcomes in patients with severe peripheral artery disease (PAD) undergoing TAVI treated with DAPT as compared to SAPT, especially amongst those with higher PAD severity [37]. While PAD intervention is necessarily concomitant to transfemoral TAVI, the timing of PCI is debated and the risk of increased access-site bleeding with DAPT should be taken into account when deciding on staging PCI before or after TAVI [38,39].

Additionally, DAPT or OAC are sometimes used after aortic valve-in-valve (ViV) interventions, due to a higher risk of thrombotic events [40,41]. Despite the fact that no RCT has been conducted in this context, observational evidence has reported a lower rate of stroke and a higher rate of minor bleeding in patients treated with DAPT, as compared to SAPT [42]. However, the difference in stroke was driven by two periprocedural events in the SAPT group, which are arguably not related to the post-procedural AT. OAC has been associated with a reduced risk of valve thrombosis after ViV [40].

Comparisons between different SAPT strategies are scarce. Compared to aspirin, clopidogrel was associated with lower cardiovascular death in an observational study [43]. On the other hand, two large RCTs compared OAC to SAPT or DAPT in patients with no previous indication for anticoagulation after TAVI [27,29]. The GALILEO (Global Study Comparing a Rivaroxaban-Based Antithrombotic Strategy to an Antiplatelet-Based Strategy After TAVR to Optimize Clinical Outcomes) trial [27] compared a dual AT (DAT) with low-dose rivaroxaban plus aspirin for 3 months, followed by rivaroxaban alone, with DAPT (aspirin plus clopidogrel) for 3 months, followed by aspirin alone. It was prematurely stopped because of harm in the experimental group, where both bleeding and ischemic outcomes were superior compared to the control arm. In contrast, in the ATLANTIS (Antithrombotic Strategy to Lower All Cardiovascular and Neurologic Ischemic and Haemorrhagic Events After TAVR for Aortic Stenosis) trial (stratum 2) [29], apixaban was found not to be superior to standard antiplatelet therapy (mostly DAPT), with a similar rate of bleeding events but a reduced incidence of obstructive valve thrombosis. Both GALILEO and ATLANTIS included a CT sub-study on smaller cohorts [20,21]. In both cases, the experimental arm using a direct OAC (DOAC) showed a lower rate of RLM and/or HALT as compared to APT. The striking difference between the CT and the clinical results of these large trials remains an open question regarding the clinical impact of HALT and RLM in this setting.

Two smaller studies further supported these results by showing a trend towards lower rates of RLM and/or HALT using OAC rather than APT, but they were underpowered to detect differences in clinical outcomes [22,28]. One large observational study explored the option of withdrawing all AT, suggesting that it could reduce bleeding without increasing ischemic risk in selected patients [44].

### 2.4. Patients with Previous Indication for Anticoagulation

Between 15 and 40% of patients undergoing TAVI have an indication for anticoagulation [45,46]. In this population, OAC was shown to be superior to DAT (OAC + clopidogrel) in terms of bleeding risk as seen in cohort B of the POPular TAVI trial [30]. When comparing DOACs with VKA, although previous observational data suggested that DOACs may be associated with a higher ischemic risk [47], this has not been confirmed in subsequent RCTs. Edoxaban was found to be noninferior to VKA in terms of net adverse cardiovascular events in the ENVISAGE-TAVI AF (Edoxaban Versus Vitamin K Antagonists After TAVR in Patients with Atrial Fibrillation) trial [31]. However, bleeding was more common with edoxaban, and this prevented testing for superiority. The authors suggested that this may be linked to subtherapeutic international normalized ratio (INR) values and a higher incidence of drug discontinuation in the VKA group. The concomitant use of APT in this population may also have contributed to this result. Similarly, in the ATLANTIS trial (stratum 1) [29], apixaban was not superior to VKA for a large composite of ischemic and bleeding endpoints, and no difference was found among the individual components. Hence, the choice of OAC should be based on the underlying indication alone, with a preference for DOACs when the indication is atrial fibrillation.

Overall, it should be noted that almost all of these trials included a rather elderly population, with high baseline bleeding and thrombotic risks. The inclusion of younger patients in future studies may reduce the impact of bleeding events and shift the focus towards ischemic and hemodynamic outcomes. Since leaflet thrombosis has emerged as a mechanism of valve dysfunction, reducing SLT with OAC may reduce valve dysfunction, although observational evidence is currently discordant on this topic [48,49].

Additionally, the potential use of left atrial appendage occlusion (LAAO) to remove OAC after TAVI was tested in the WATCH-TAVR trial (WATCHMAN for Patients with AF Undergoing TAVR), which found this option to be noninferior to medical therapy [50]. However, superiority remains to be demonstrated.

Another source of uncertainty comes from the open-label design of all these RCTs. This can lead to performance and detection bias, especially when assessing partially subjective endpoints (such as bleeding classifications). Hence, double-blind RCTs should be encouraged.

### 2.5. Periprocedural Management of Anti-Thrombotic Therapy Prior to TAVI

For patients on SAPT, it should not be stopped before TAVI, and it can be initiated even before the procedure if the patient is not already treated [51]. For patients on OAC, periprocedural continuation has been shown to be noninferior to interruption in a recent RCT (82% of patients were on DOAC, 18% on VKA) but with a higher risk of minor bleeding complications [52]. Intraprocedural unfractionated heparin (UFH) to obtain an activated clotting time > 250 s and full protamine reversal at the end of the procedure are standard in most centers [53].

### 2.6. Future Directions

**Figure 3:**
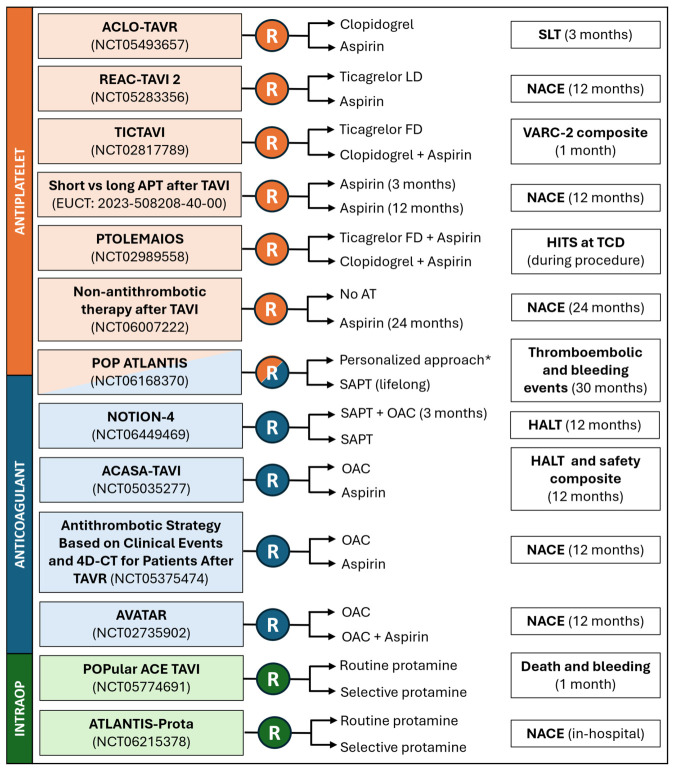
Ongoing RCT assessing AT after TAVI. * see text for details. Abbreviations: APT: antiplatelet therapy; AT: antithrombotic therapy; CT: computed tomography; FD: full dose; HALT: hypo-attenuated leaflet thickening; HITS: high-intensity transient signals; LD: loading dose; NACE: net adverse clinical events; OAC: oral anticoagulation; SAPT: single antiplatelet therapy; SLT: subclinical leaflet thrombosis; TAVI: transcatheter aortic valve implantation; TAVR: transcatheter aortic valve replacement; TCD: transcranial Doppler; VARC-2: valve academic research consortium-2.

Several ongoing studies will help address some unanswered questions, including composition and duration of the optimal SAPT strategy, the role of DAPT and the combination of APT and OAC (Figure 3). Interesting approaches are proposed by the NOTION (Nordic Aortic Valve Intervention)-4 trial (NCT06449469) [54], which will compare a 3-month DAT (OAC plus aspirin, followed by aspirin) versus aspirin for prevention of HALT on serial CT scans, and by the POP ATLANTIS trial (NCT06168370), which will test lifelong SAPT versus a personalized approach based on a 3-month CT scan: if no SLT is detected, SAPT is stopped, unless another indication is present; if SLT is found, apixaban will be given for 6 months and either continued or discontinued depending on the persistence of SLT after 6 months. Total AT withdrawal is also being investigated (NCT06007222).

Further data will be needed to assess the best AT regimen after TAVI in special conditions (recent PCI or ViV), the impact of SLT on clinical outcomes, and the role of anti-XIa inhibitors and LAAO. Additional factors may influence the optimal AT strategy, such as valve underexpansion or younger patient age. In severely underexpanded valves, OAC may be used to prevent HALT, while in young patients at low bleeding risk, the use of OAC could be beneficial in improving valve durability. Given the diverging results of currently available studies and the high number of ongoing RCTs, quantitative synthesis through updated meta-analyses will also be needed to reach more robust conclusions on these topics.

## 3. Transcatheter Mitral and Tricuspid Valve Interventions

### 3.1. Transcatheter Mitral Valve Interventions

Mitral-transcatheter edge-to-edge repair (M-TEER) is an established treatment strategy for patients with severe mitral regurgitation deemed unsuitable for cardiac surgery [1]. Current antithrombotic strategies derive from the regimens adopted in clinical trials, since no RCT has directly investigated AT after M-TEER. In the EVEREST (Endovascular Valve Edge-to-Edge Repair Study) trial, 1-month DAPT with aspirin and clopidogrel followed by aspirin for 6–12 months was recommended [55]. More recently, the COAPT (Cardiovascular Outcomes Assessment of the MitraClip Percutaneous Therapy for Heart Failure Patients With Functional Mitral Regurgitation) trial protocol recommended either SAPT or DAPT in patients who were not receiving OAC [56]. On this basis, current European guidelines recommend SAPT in patients without an indication for OAC [1] (Table 1).

Transcatheter mitral valve replacement (TMVR) has emerged as an alternative option for patients with native mitral valve disease who are considered at prohibitive surgical risk, as well as patients with degenerated bioprosthetic valves (valve-in-valve [ViV]), failed annuloplasty rings (valve-in-ring [ViR]), and severe mitral annular calcification (valve-in–mitral annular calcification [ViMAC]) [1,57]. However, TMVR is associated with a risk of device-related thrombosis (DRT), mainly due to the low velocity of transmitral flow. DRT has been reported in approximately 6–9% of TMVR procedures [57,58], with the highest incidence occurring within the first three months, especially in the setting of suboptimal AT [59].

For surgical MVR, VKA for 3–6 months followed by aspirin is recommended. For TMVR, evidence to guide the optimal AT remains limited, resulting in substantial heterogeneity in post-procedural management. This variability is further compounded by the high prevalence of concomitant indications for anticoagulation, particularly atrial fibrillation (AF), within this population. Thus, both VKA and DOACs have been employed, either as monotherapy or in combination with aspirin [59]. In a single-center registry of 130 patients treated with ViV, ViR or ViMAC using SAPIEN XT/3 (Edwards Lifesciences), the one-year cumulative incidence of DRT reached 11.1%, with most events occurring early after the procedure despite anticoagulation with a VKA (target INR 2.0–3.0) combined with low-dose aspirin [60]. More recently, a comparative study of 156 patients undergoing TMVR demonstrated that DOACs were associated with a lower risk of bleeding compared with VKA (9% vs. 35%), without an increase in thrombotic events [59].

Concerning TMVR in native mitral regurgitation, in a small cohort of patients treated with transfemoral transseptal TMVR using the Intrepid system (Medtronic), no clinically significant DRT was observed at 30 days. However, DRT was reported in 3.4% of patients at one year of follow-up [61]. In the ENCIRCLE (Transcatheter Mitral Valve Replacement via Transseptal Access) trial, all patients were treated with a SAPIEN M3 device (Edwards Lifesciences) and received oral anticoagulation for at least six months (either DOACs or VKA). Clinically significant DRT occurred in 2.3% and 6.7% of patients at 30 days and one year, respectively, and did not differ between DOAC (7% at one-year) and VKA (6%). Importantly, in patients with inadequate anticoagulation (defined as a gap greater than 1 day in anticoagulation or INR < 2.5 if taking warfarin), rates of clinical or subclinical thrombosis were as high as 20.9% [62].

Current guidelines recommend at least six months of oral anticoagulation (either DOACs or VKA) following TMVR in patients without additional indications for anticoagulation, and lifelong therapy in those with a pre-existing indication (Table 1) [1]. Whether long-term anticoagulation should be offered to all patients undergoing TMVR is currently unknown.

### 3.2. Transcatheter Tricuspid Valve Interventions

Transcatheter therapies for tricuspid valve disease have recently emerged as a promising alternative to treat patients with symptomatic severe tricuspid regurgitation (TR), although their impact on hard clinical endpoints is still under investigation [63].

Tricuspid TEER is an established therapeutic option for patients with severe tricuspid regurgitation. In the TRILUMINATE (Trial to Evaluate Treatment With Abbott Transcatheter Clip Repair System in Patients With Moderate or Greater Tricuspid Regurgitation) trial, which compared T-TEER with optimal medical therapy, DRT was reported in only two patients (0.7%) [64]. Moreover, no cases of DRT were reported in a large real-world post-market prospective registry [65]. It should be noted that this population is frequently on long-term OAC due to the high prevalence of AF. Therefore, additional antithrombotic therapy is generally not required [64]. Currently, long-term SAPT is recommended unless there is a pre-existing indication for OAC (Table 1) [1].

**Figure 4:**
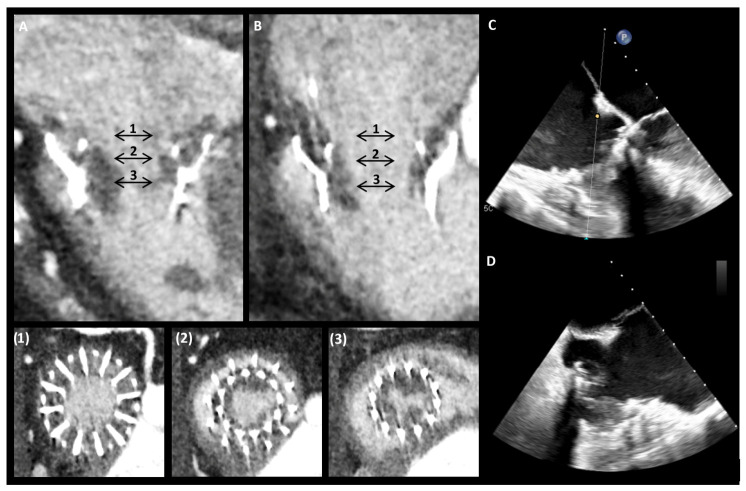
Clinically relevant thrombosis of a transcatheter tricuspid prosthesis. Computed tomography showed diffuse thrombotic involvement of the prosthesis ((**A**): long-axis, 4-chamber view; (**B**): long-axis, 3-chamber view; short-axis sections are reported in Figures (**1**)–(**3**)). Thrombus was also detectable by transesophageal echocardiography ((**C**): mid-esophageal view, 0°; (**D**): mid-esophageal view, 155°).

Compared to T-TEER, transcatheter tricuspid valve replacement (TTVR) has an increased risk of DRT. The thrombogenic potential of this procedure is attributed to multiple factors, including the slow blood flow within the right heart chambers and the large surface area of the prosthetic tricuspid valves. Subclinical leaflet thrombosis can develop early [66]. It has been reported in up to 27% of cases and is associated with reduced functional improvement [67]. Concerning clinical thrombosis, no device-related pulmonary embolism was reported at one year in the TRISCEND (Transcatheter Valve Replacement in Severe Tricuspid Regurgitation) II study, where patients treated with the EVOQUE system (Edwards Lifesciences) received OAC (either a VKA or a DOAC) in addition to SAPT with aspirin [68]. However, data regarding DRT were not provided. Device design and materials may have an impact on thrombotic and bleeding events. In a multicenter study analyzing 126 patients treated with TTVR using the LuX-Valve system (Jenscare Biotechnology), all patients received OAC for a minimum of six months. At one year of follow-up, only one case of DRT (0.8%) was reported [69]. An example of device thrombosis is reported in Figure 4.

Nevertheless, the high hemorrhagic risk associated with these procedures should be considered when choosing the optimal AT. In TRISCEND, 15.4% of patients experienced a severe bleeding event, and similar numbers were reported using LuX-Valve [68,69].

AT with at least 6 months of OAC is the currently recommended regimen following TTVR (Table 1) [1]. When choosing between DOAC and VKA, it is reasonable to maintain the pre-existing OAC also after TTVR. A close follow-up using multimodal imaging after TTVR is recommended, as echocardiography may capture increasing gradients related to HALT, but CT scanning is more sensitive for detecting subclinical thrombosis and thus informing AT management [70].

For both mitral and tricuspid interventions, dedicated RCTs are needed to assess the optimal AT and the role of routine CT guidance during follow-up.

## 4. Patent Foramen Ovale and Atrial Septal Defect Closure

Evidence guiding long-term AT after transcatheter patent foramen ovale (PFO) and atrial septal defect closure is limited. Thus, current clinical practice is primarily based on expert consensus and on antithrombotic strategies extrapolated from pivotal PFO closure trials.

Across these trials, post-procedural AT typically involved DAPT for 1–6 months followed by SAPT to prevent device-related thrombosis (DRT) during endothelization, which generally occurs within 3–6 months. However, additional factors such as device composition, surface characteristics, and nickel hypersensitivity can all delay endothelization [71,72,73,74]. However, DRT is a rare event in contemporary RCTs, with isolated cases reported in the CLOSE (Patent Foramen Ovale Closure or Anticoagulants versus Antiplatelet Therapy to Prevent Stroke Recurrence), REDUCE (Patent Foramen Ovale Closure or Antiplatelet Therapy for Cryptogenic Stroke) (≈0.5%), and RESPECT (Randomized Evaluation of Recurrent Stroke Comparing PFO Closure to Established Current Standard of Care Treatment) (≈0.2%), and none in DEFENSE-PFO (Device Closure Versus Medical Therapy for Cryptogenic Stroke Patients With High-Risk Patent Foramen Ovale) [71,72,73,74] (Table 4). Moreover, observational data suggest that 3 months of DAPT offers no significant advantage over SAPT, as shown in a large cohort of 1532 patients in whom dual therapy did not reduce ischemic or bleeding events over a 5-year follow-up [75]. Consistent with these findings, the CANOA (Clopidogrel for the Prevention of New-Onset Migraine Headache Following Transcatheter Closure of Atrial Septal Defects) trial showed that short-term clopidogrel after atrial septal defect closure did not affect ischemic or bleeding outcomes (but reduced migraine burden) [76].

Overall, modern PFO occluders are associated with a low thrombotic risk below 1% with standard AT; however, consensus recommendations remain heterogeneous. European and interventional guidelines favor an individualized strategy with short-term DAPT (1–6 months) followed by SAPT for at least 5 years and discourage routine long-term anticoagulation, whereas American guidelines support lifelong AT [77,78] (Table 1). Ongoing studies, including the HALTI trial (Discontinuation of Antithrombotic Treatment Following PFO Closure in Young Patients With Cryptogenic Stroke, NCT04475510), are expected to clarify the safety of complete antiplatelet discontinuation after PFO closure in carefully selected low-risk patients.

## 5. Left Atrial Appendage Occlusion

Transcatheter LAAO is an established procedure for stroke prevention in patients with non-valvular atrial fibrillation, particularly in those at increased bleeding risk or with contraindications to long-term OAC. The optimal post-procedural AT remains incompletely defined, particularly given that DRT occurs in approximately 3–5% of patients and is associated with a 4- to 5-fold increased risk of ischemic stroke or systemic embolism during the endothelization phase [79].

Early RCTs were conducted before the widespread adoption of DOACs, and their protocols have influenced regulatory approvals and guideline recommendations (Table 1). However, real-world evidence showed that fewer than 40% of patients are discharged on the recommended combinations of anticoagulant and antiplatelet therapy [80].

### 5.1. Patients Without Contraindication to Anticoagulation

In patients eligible for short-term anticoagulation, AT strategies post-LAAO were initially based on pivotal randomized trials [81,82], which mandated warfarin plus aspirin for 45 days, followed by DAPT for up to 6 months, and lifelong aspirin thereafter (Table 5). In a pooled analysis of trials using the Watchman device (Boston Scientific), including 1739 patients, DRT occurred in 3.74% of cases and was detected both early and late after implantation [83]. DRT was associated with a significantly increased risk of stroke or systemic embolism (adjusted rate ratio 3.55, 95% CI 2.18–5.79), although the majority of ischemic events (86.6%) occurred in patients without documented DRT. Another analysis of these trials demonstrated that major bleeding events were most frequent during the early postprocedural phase, particularly when patients were receiving combined warfarin and aspirin therapy and declined substantially after de-escalation to single antiplatelet therapy [84].

Real-world data have further questioned the safety advantage of combining OAC with antiplatelet therapy. In a registry involving more than 30,000 patients, the addition of aspirin to anticoagulation did not significantly reduce ischemic events but was associated with higher rates of major bleeding during early follow-up [93]. These findings, together with improvements in device design and sealing properties, have contributed to a progressive shift toward simplified regimens.

DOAC-based strategies have increasingly replaced vitamin K antagonists in contemporary practice. Prospective studies evaluating DOAC plus aspirin for 45 days have reported DRT rates between 1.7% and 3.0%, with major bleeding rates below 3% at one year [86]. These results led to regulatory approval of DOAC-based regimens for newer-generation devices. Beyond standard-dose anticoagulation, low-dose DOAC strategies have gained interest. In a non-randomized cohort, low-dose DOACs were associated with a 70–90% relative reduction in the composite endpoint of DRT, thromboembolic events, and major bleeding compared with conventional regimens [94].

Randomized evidence, although limited, supports this approach. In a prematurely terminated randomized trial comparing low-dose apixaban (2.5 mg twice daily) with 3-month DAPT, the composite endpoint of major bleeding, thromboembolic events, and DRT occurred in 4.6% of patients receiving apixaban versus 21.7% in the DAPT group (hazard ratio 0.18; 95% confidence interval 0.04–0.84) [90]. Major and minor bleeding combined was also significantly reduced (4.6% vs. 28.3%).

### 5.2. Patients with Contraindication to Anticoagulation

In patients with formal contraindications to OAC, LAAO is commonly used as an alternative to lifelong anticoagulation, with short-term DAPT followed by SAPT as the predominant postprocedural regimen, particularly in Europe. In large registries, DAPT was prescribed in 60–75% of patients, in line with European device labeling and Food and Drug Administration approvals supporting DAPT after Amplatzer Amulet (Abbott) implantation and as a 45-day alternative to OAC after Watchman FLX [95,96].

Despite its widespread adoption in patients with higher bleeding risk, the safety and efficacy of DAPT after LAAO remain uncertain, as consistent bleeding reduction compared with OAC has not been demonstrated. In the global Amulet registry, major bleeding occurred in 8.4% of patients on DAPT versus 4.1% on OAC alone, while a propensity-matched analysis reported a higher rate of DRT with antiplatelet therapy compared with OAC (3.1% vs. 1.4%; *p* = 0.014) [96,97]. Overall, interpretation is limited by the observational design, heterogeneous population, and selection bias.

In patients with extreme bleeding risk who are unable to tolerate either short-term OAC or DAPT, further de-escalation to SAPT has been adopted, although evidence remains limited and controversial. In the EWOLUTION registry, 7.0% of patients received SAPT, with a reported DRT rate of 3.8%, comparable to other antithrombotic regimens [95]. Similarly, in the Amulet registry, 23.0% of patients were discharged on SAPT, with DRT occurring in 2.2%, contributing to a low overall DRT rate of 1.7% [96]. However, these data are limited to small, observational cohorts and available registry data suggest a higher risk of DRT with complete antithrombotic withdrawal, indicating that SAPT should be restricted to carefully selected patients after individualized multidisciplinary risk–benefit assessment.

More recently, two RCTs have tested LAAO in different populations. In CLOSURE-AF, which enrolled 888 patients at particularly high thromboembolic and hemorrhagic risk (mean CHA_2_DS_2_-VASc 5.2; HAS-BLED 3.0), LAAO failed to demonstrate noninferiority versus physician-directed best medical therapy and was associated with a numerically higher incidence of the primary composite endpoint of stroke, systemic embolism, major bleeding, or cardiovascular/unexplained death [91]. Importantly, the excess risk was largely driven by early procedural and periprocedural complications, without a clear downstream bleeding advantage sufficient to offset these hazards. By contrast, the CHAMPION-AF trial evaluated a lower-risk and more selected population of 3000 patients who were suitable candidates for long-term anticoagulation (mean CHA_2_DS_2_-VASc 3.5; HAS-BLED 1.3) [92]. In this trial, device-based LAAO was noninferior to non-vitamin K antagonist oral anticoagulant therapy for the composite of cardiovascular death, stroke, or systemic embolism at 3 years, while significantly reducing non-procedure-related bleeding.

Taken together, CLOSURE-AF and CHAMPION-AF suggest that the clinical value of LAAO is highly dependent on baseline patient risk, comparator therapy, and procedural safety. LAAO may represent a reasonable alternative to anticoagulation in carefully selected patients with acceptable procedural risk, but its benefit is far less certain in frail, high-bleeding-risk populations in whom early complications and the need for adjunctive antithrombotic therapy may negate the expected advantage.

**Figure 5:**
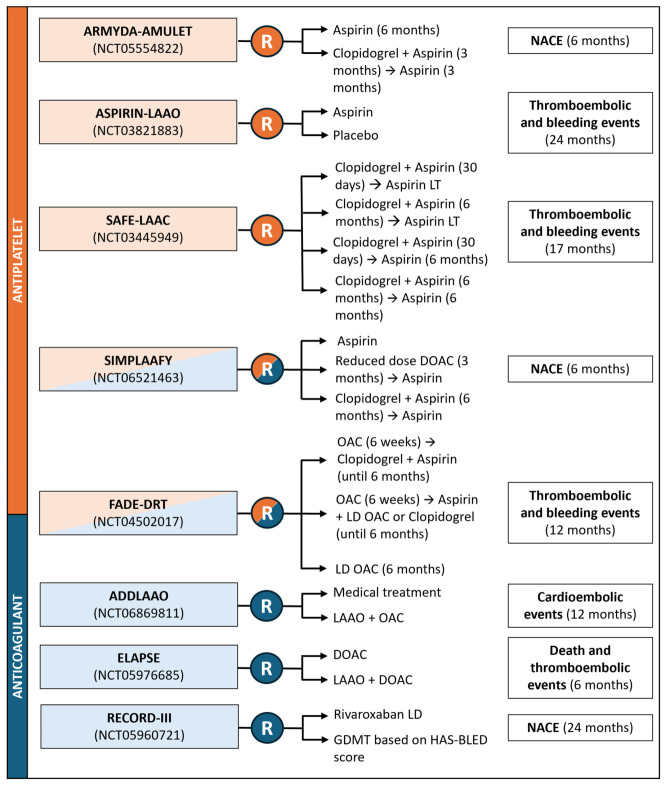
Ongoing RCTs assessing AT after LAAO. Abbreviations: (D)OAC, (direct) oral anticoagulant; GDMT: guideline-directed medical therapy; LAAO: left atrial appendage occlusion; LD, low dose; NACE: net adverse cardiovascular events; RCTs, randomized controlled trials.

Numerous ongoing randomized trials are expected to further refine the optimal AT following LAAO, mostly focusing on SAPT and low-dose DOAC (Figure 5). Since the risk profile of patients undergoing LAAO can be very different—ranging from a very high thrombotic risk to a very bleeding risk, a tailored approach focused on each patient’s dominant risk profile is likely to be the future of this field.

## 6. Conclusions

Considerable gaps remain in our understanding of the optimal AT after structural heart interventions (*Central illustration*). While post-TAVI antithrombotic strategies can benefit from randomized studies, robust evidence is still scarce for most other transcatheter procedures. Device-related thrombosis is not uncommon, but the bleeding risk is increased by aggressive AT. Future research should therefore focus on procedure-specific and patient-centered strategies, aiming to define individualized antithrombotic approaches that achieve an optimal balance between thrombotic protection and bleeding risk.

## Figures and Tables

**Table 1:** Current guidelines’ recommendations on AT after structural heart interventions.

Scenario	Recommendation		European Guidelines		US Guidelines	
	Therapy	Timing/Condition	Class	LOE	Class	LOE
TAVI						
TAVI with no previous OAC indication	ASA	12 months	I	A		
	ASA	Long term	IIa	C	IIa	B-R
	DAPT		III	B		
	OAC		III	A		
	DAPT (A + C)	3–6 months, if low bleeding risk			IIb	B-R
	VKA	3 months, if low bleeding risk			IIb	B-R
	Rivaroxaban LD +ASA				III	B-R
TAVI with no previous OAC indication and recent PCI (<3 months)	DAPT (A + C)	1–6 months	Consensus			
TAVI with previous OAC indication	OAC	Long term	I	B	I	L-D
TAVI with previous OAC indication and recent PCI (<3 months)	OAC, + Clopidogrel for 1–6 months + ASA for 0–30 days		Consensus			
Clinical valve thrombosis	OAC (VKA)	before considering reintervention	I	B	IIa	B-NR
Subclinical valve thrombosis	OAC	if HALT/RLM leads to elevated gradients, at least until resolution	IIa	B		
Mitral and tricuspid interventions						
M-TEER or T-TEER with no previous OAC indication	SAPT	Long-term	No class			
TMVR or TTVR with no previous OAC indication	OAC	≥6 months	No class			
In any case of previous OAC indication	OAC	Long-term	No class			
PFO						
After PFO closure	DAPT	1–6 months	Consensus			
	SAPT	At least 5 years	Consensus			
LAAO						
LAAO with contraindication to OAC	DAPT (A + C) for 1–6 months → ASA long-term		Consensus			
LAAO with no contraindication to OAC	OAC (VKA or DOAC) plus ASA for 45 days * → DAPT (A + C) up to 6 months → ASA long-term		Consensus			

ASA: Acetylsalicylic Acid; DAPT: Dual Antiplatelet Therapy; (D)OAC: (Direct) Oral Anticoagulant; LAAO: Left Atrial Appendage Occlusion; LD: Low Dose; LOE: Level of Evidence; M-TEER: Mitral Transcatheter Edge-to-Edge Repair; PCI: Percutaneous Coronary Intervention; PFO: Patent Foramen Ovale; SAPT: Single Antiplatelet Therapy; T-TEER: Tricuspid Transcatheter Edge-to-Edge Repair; TAVI: Transcatheter Aortic Valve Implantation; TMVR: Transcatheter Mitral Valve Replacement; TTVR: Transcatheter Tricuspid Valve Replacement; VKA: Vitamin K Antagonist. * If a significant (>5 mm) residual leak persists at 45-day transesophageal echocardiography, OAC combined with aspirin should be prolonged until adequate appendage sealing is achieved. Recommendations are reported in green for class I, yellow for class IIa, orange for class IIb and red for class III.

**Table 2:** Randomized studies testing antithrombotic strategies after TAVI. Only the most important results for each study are reported. Results are reported as treatment arm vs. control arm.

Study	Design	Patients (N)	Age (Mean)	Sex (Males)	Valves Used	Treatment	Control	FU (Months)	Key Results	
									Bleeding	Ischemic
No previous indication for OAC										
Testing DAPT										
**Ussia et al.** [23], 2011	OL	79	81	46%	SEV	DAPT (A + C) for 3 months → SAPT (A)	SAPT (A)	6	-Major bleeding: 5% vs. 3% (*p* = 0.61)	-No differences in any ischemic outcome
**SAT-TAVI** [24], 2014	OL	120	81	37%	BEV	DAPT (A + C or A + T) for 6 months	SAPT (A)	6	-Any bleeding: 15% vs. 10% (*p* not reported) -Any VC: 13.3% vs. 5.0% (*p* < 0.05)	-No differences in any ischemic outcome
**ARTE** (NCT01559298) [25], 2017	OL	222	79	63%	BEV	DAPT (A + C) for 3 months	SAPT (A)	3	-Major or life-threatening bleeding: 10.8% vs. 3.6% (*p* = 0.038)	-No differences in any ischemic outcome
**POPular TAVI—cohort A** (NCT02247128) [26], 2020	OL	665	80	51%	Any	DAPT (A + C) for 3 months → SAPT (A)	SAPT (A)	12	-All bleeding: 26.6% vs. 15.1% (*p* = 0.001) -Major, disabling or life-threatening bleeding: 10.8% vs. 5.1% (*p* not reported)	-No differences in any ischemic outcome
Testing OAC										
**GALILEO** (NCT02556203) [27], 2019	OL	1644	80	51%	Any	DAT (rivaroxaban 10 mg+ A) for 3 months → OAC (rivaroxaban 10 mg)	DAPT (A + C) for 3 months → SAPT (A)	17	-Major, disabling or life-threatening bleeding: 4.3 vs. 2.8/100 p-y (*p* = 0.08) -Major bleeding: 2.8 vs. 1.4/100 p-y (*p* not reported)	-Death or thromboembolic events: 9.8 vs. 7.2/100 p-y (*p* = 0.04)
**GALILEO-4D** (NCT02833948) [20], 2020	OL	231	79	53%	Any	DAT (rivaroxaban 10 mg+ A) for 3 months → OAC (rivaroxaban 10 mg)	DAPT (A + C) for 3 months → SAPT (A)	3	\	-RLM ≥ 3: 2.1% vs. 10.9% (*p* = 0.01) -SLT: 32.4% vs. 12.4% (*p* not reported)
**LRT 2.0** (NCT03557242) [28], 2021	OL	94	73	70%	Any	DAT (VKA + A) for 1 month → OAC (VKA)	SAPT (A)	1	-No differences in any bleeding endpoint	-Composite of HALT, RLM, hemodynamic dysfunction, stroke and TIA: 7.0% vs. 26.5% (*p* = 0.014). -HALT: 4.7% vs. 16.3% (*p* = 0.07) -RLM ≥ 2: 2.3% vs. 10.4% (*p* = 0.12) -Hemodynamic dysfunction: 2.3% vs. 10.0% (*p* = 0.13) -HALT: 0.0% vs. 4.0% (*p* = 0.18)
**ATLANTIS—stratum 2** (NCT02664649) [29], 2022	OL	1049	82	52%	Any	DOAC (apixaban 2.5 or 5 mg)	Standard antiplatelet therapy (mostly DAPT with A + C)	12	-Major, disabling, fatal or life-threatening bleeding: 7.8% vs. 7.3% (*p* = NS)	-Obstructive valve thrombosis: 1.1% vs. 6.1% (*p* not reported) -No differences in any clinical ischemic outcome
**ATLANTIS-** **4D-CT** (NCT02664649) [21], 2022	OL	762	82	46%	Any	DOAC (apixaban 2.5 or 5 mg)	Standard of care	3–6	\	-RLM ≥ 3 or HALT ≥ 4: 8.9% vs. 13.0% (*p* = 0.037) -Significant interaction with indication for anticoagulant: 8.7% vs. 15.9% (*p* = 0.011) in pts with no indication; 9.5% vs. 5.5% (*p* = 0.28) in those with indication.
**ADAPT-TAVR** (NCT03284827) [22], 2022	OL	229	80	48%	Any	DOAC (edoxaban 30 or 60 mg)	DAPT (A + C) for 6 months	6	-Any bleeding: 11.7% vs. 12.7% (*p* = 0.82)	-HALT ≥ 1: 9.8% vs. 18.4% (*p* = 0.08) -RLM ≥ 3: 2.9% vs. 7.3% (*p* = 0.15) -No differences in any clinical or imaging ischemic outcome
Previous indication for OAC										
**POPular TAVI—Cohort B** (NCT02247128) [30], 2020	OL	313	81	54%	Any	DAT (OAC + clopidogrel) for 3 months → OAC alone	OAC alone	12	-All bleeding: 34.6% vs. 21.7% (*p* = 0.01) -Major, disabling or life-threatening bleeding: 16.7% vs. 8.9% (*p* not reported)	-No differences in any ischemic outcome
**ENVISAGE-** **TAVI AF** (NCT02943785) [31], 2021	OL	1426	82	52%	Any	DOAC (edoxaban 30 or 60 mg)	VKA	18	-Major bleeding: 9.7 vs. 7.0/100 p-y (*p* = 0.93 for non-inferiority) -GI bleeding: 5.4 vs. 2.7/100 p-y (*p* not reported)	-No differences in any ischemic outcome -NACE: 17.3 vs. 16.5/100 p-y (*p* = 0.01 for non-inferiority)
**ATLANTIS—stratum 1** (NCT02664649) [29], 2022	OL	451	82	45%	Any	DOAC (apixaban 2.5 or 5 mg)	Standard of care (mostly VKA)	12	-Major, disabling, fatal or life-threatening bleeding: 10.3% vs. 11.4% (*p* = NS)	-No differences in any ischemic outcome

BEV: balloon-expandable valve; DAPT: dual antiplatelet therapy; (D)AT: (dual) antithrombotic therapy; DOAC: direct oral anticoagulant; FU: follow-up; GI: gastro-intestinal; HALT: hypoattenuated leaflet thickening; NACE: net adverse clinical events; OL: open-label; RLM: restricted leaflet motion; p-y: person-years; SAPT: single antiplatelet therapy; SEV: self-expanding valve; SLT: sublinical leaflet thrombosis; TIA: transient ischemic attack; VC: vascular complication; VKA: vitamin K antagonist.

**Table 3:** Bioengineering composition of main commercial and experimental bioprosthetic valves for aortic, mitral and tricuspid valve replacement. The definition of SEV or BEV is not fully translatable from the aortic setting to the mitral/tricuspid one, so it should be merely considered as an indication of the valve releasing mechanism.

	Valve Model	Stent Frame	Leaflet (Pericardium)	Releasing Mechanism
Aortic	SAPIEN 3/3 Ultra/3 ultra Resilia (Edwards Lifesciences, Irvine, CA, USA)	Cobalt-chromium	Bovine	BEV
	Myval/Myval Octacor/Octapro/Octapro Plus (Meril, Vapi, Guyarat, India)	Nickel-cobalt	Bovine	BEV
	Evolut R/PRO/PRO+/FX/FX+ (Medtronic, Minneapolis, MN, USA)	Nitinol	Porcine	SEV
	Portico/Navitor/Navitor Vision (Abbott, Menlo Park, CA, USA)	Nitinol	Bovine	SEV
	Allegra (NewValveTechnology, Hechingen, Germany)	Nitinol	Bovine	SEV
	Hydra Valve (SMT, Mumbai, India)	Nitinol	Bovine	SEV
	VitaFlow/VitaFlow Liberty (MicroPort, Shanghai, China)	Nitinol	Bovine	SEV
	JenaValve Trilogy (Edwards Lifesciences, Irvine, CA, USA)	Nitinol	Porcine	SEV
	J-Valve (JC Medical, Burlingame, CA, USA)	Nitinol	Bovine	SEV
Mitral	Tendyne (Abbott, Menlo Park, CA, USA)	Nitinol	Porcine	SEV
	Intrepid (Medtronic, Minneapolis, MN, USA)	Nitinol	Bovine	SEV
	AltaValve (4C Medical Technologies, Minneapolis, MN, USA)	Nitinol	Bovine	SEV
	SAPIEN M3 (Edwards Lifesciences, Irvine, CA, USA)	Cobalt-chromium (valve) and nitinol (docking frame)	Bovine	BEV
	Innovalve (Edwards Lifesciences, Irvine, CA, USA)	Nitinol	Bovine	SEV
	EVOQUE Eos (Edwards Lifesciences, Irvine, CA, USA)	Nitinol	Bovine	SEV
	ValvSync (Symbios, Petach Tikva, Israel)	Not disclosed	Not disclosed	Balloon-mediated anchoring
	Tioga (Tioga Cardiovascular, Los Gatos, CA, USA)	Nitinol	Not disclosed	SEV
	Saturn (Innovheart, Milan, Italy/Boston, MA, USA)	Nitinol	Bovine	SEV
	Cardiovalve (Venus Medtech, or Yehuda, Israel)	Nitinol	Bovine	SEV
	Cephea (Abbott, Menlo Park, CA, USA)	Nitinol	Bovine	SEV
	HighLife (HighLife SAS, Paris, France)	Nitinol	Bovine	SEV
Tricuspid	Evoque (Edwards Lifesciences, Irvine, CA, USA)	Nitinol	Bovine	SEV
	Intrepid (Medtronic, Minneapolis, MN, USA)	Nitinol	Bovine	SEV
	Lux-Valve Plus (Jenscare, Ningbo, China)	Nitinol	Bovine	SEV
	Trisol (Trisol Medical, Yokneam, Israel)	Nitinol	Bovine	SEV
	Topaz (TRiCares, St. Louis Park, MN, USA)	Nitinol	Bovine	SEV
	Cardiovalve (Venus Medtech, or Yehuda, Israel)	Nitinol	Bovine	SEV
	VDyne (VDyne, Maple Grove, MN, USA)	Nitinol	Bovine	SEV
	MonarQ (InQB8 Medical Technologies, Burlington, MA, USA)	Nitinol	Bovine	SEV

BEV: balloon-expandable valve; SEV: self-expandable valve.

**Table 4:** Patent foramen ovale trials on cryptogenic stroke.

Study	Patients (n)	Device	AT	AT Duration	Follow-Up, Years	Stroke	Device Thrombosis
CLOSE (NCT00562289) [71], 2017	660	Multiple	DAPT (A + C) for 3 months → SAPT (A or C)	>5 years	5.3	0%	0%
REDUCE (NCT00738894) [72], 2017	664	Helex/GSO	SAPT (C for 3 days → A or C)	>3 years	3.2	1.4%	0.5%
RESPECT (NCT00465270) (long-term results) [73], 2017	980	Amplatzer	DAPT (A + C) for 1 month, → SAPT (A for 5 months → A or C)	6 months or >5 years	5.9	3.6%	3.3%
DEFENSE-PFO (NCT01550588) [74], 2018	120	Amplatzer	DAPT (A + C) or SAPT (A) or OAC	6 months or >2.5 years	2.8	0%	NR

Abbreviations: A, aspirin; AT, antithrombotic therapy; C, clopidogrel; DAPT, dual antiplatelet therapy; GSO, GORE septal occluder; NR: not reported; OAC, oral anticoagulation; SAPT, single antiplatelet therapy.

**Table 5:** Left atrial appendage occlusion trials.

Study	Patients (N)	Device	AT Regimen	Follow-Up, Months	Major Bleeding	Ischemic Stroke	DRT
PROTECT-AF (NCT00129545) [81], 2014	463	Watchman	DAT (VKA + A) for 45 days → DAPT (A + C) for up to 6 months * → SAPT (A)	46	0.2% ^†^	1.4%	NR
PREVAIL (NCT01182441) [82], 2014	269	Watchman	DAT (VKA + A) for 45 days → DAPT (A + C) for up to 6 months * → SAPT (A)	18	0.4% ^†^	1.9%	NR
ASAP (NCT00851578) [85], 2013	150	Watchman	DAPT (A + C) for 6 months → SAPT (A)	14	0.6% ^†^	1.7%	0.7%
PINNACLE-FLX (NCT02702271) [86], 2021	400	Watchman FLX	DAT (DOAC + A) for 45 days → DAPT (A + C) for up to 6 months * → SAPT (A)	12	7.75%	2.6%	1.75%
AMULET IDE (NCT02879448) [87], 2021	903	Amulet	DAPT (A + C) or DAT (A + OAC), for 45 days → DAPT (A + C) for up to 6 months * → SAPT (A)	18	0.3%	1.5%	3.3%
	896	Watchman 2.5	DAT (VKA + A) for 45 days → DAPT (A + C) for up to 6 months * → SAPT (A)		0.7%	1.4%	4.5%
SWISS-APERO (NCT03399851) [88], 2021	111	Amulet	DAPT (A + C) or OAC for 3 months → SAPT (A) for up to 12 months	1.5	7.2%	1.8%	3.7%
	110	Watchman/FLX			1.8%	0.0%	9.9%
ADRIFT (NCT03273322) [89], 2020	33 (DAPT)	Amulet and Watchman	DAPT (A + C) vs. OAC (Rivaroxaban RD) for 3 months	3	27.3%	0.0%	0.0%
	34 (Rixaroxaban 15 mg)				11.4%	0.0%	0.0%
	37 (Rixaroxaban 10 mg)				24.3%	2.7%	0.0%
ADALA (NCT05632445) [90], 2024	44 (low dose OAC)	Multiple (mainly Amulet and Watchman FLX)	DAPT (A + C) vs. OAC (Apixaban LD) for 3 months → SAPT (A)	3	4.6%	0.0%	0.0%
	46 (DAPT)				13%	0.0%	8.7%
CLOSURE-AF (NCT03463317) [91], 2026	888	Multiple (mainly Amulet and Watchman FLX)	DAPT (A + C) for 3 months → aspirin up to 6 months (if no significant leak or DRT at TEE)	36	15.7%	NR	3.1%
CHAMPION-AF (NCT04394546) [92], 2026	3000	Watchman FLX	DOAC + aspirin, DOAC monotherapy or DAPT for 3 months → SAPT (A or C)	36	5.1%	3.2%	4.8%

Abbreviations: A, aspirin; ATT, antithrombotic; C, clopidogrel; DAPT, dual antiplatelet therapy; DAT, dual antithrombotic therapy; DOAC, direct oral anticoagulant; DRT: device-related thrombosis; LD, low dose; NR, not reported; OAC, oral anticoagulation; RCTs, randomized controlled trials; RD, reduced dose; SAPT, single antiplatelet therapy; TEE: trans-esophageal echocardiography; VKA: vitamin-K antagonist. * If, at the 45-day transesophageal echocardiographic assessment, no device-related thrombus was detected and the peri-device leak measured ≤5 mm; ^†^ Hemorrhagic stroke.

## Data Availability

No new data were created or analyzed in this study. Data sharing is not applicable to this article.

## Abbreviations

AF	atrial fibrillation
ASA	acetylsalicylic acid
AT	antithrombotic therapy
BEV	balloon-expandable valve
CT	computed tomography
DAT	dual antithrombotic therapy
DAPT	dual antiplatelet therapy
DOAC	direct oral anticoagulant
DRT	device-related thrombosis
FU	follow-up
GI	gastrointestinal
HALT	hypoattenuated leaflet thickening
INR	international normalized ratio
LAAO	left atrial appendage occlusion
LD	low dose
LOE	level of evidence
NACE	net adverse clinical events
OAC	oral anticoagulation
PAD	peripheral artery disease
PCI	percutaneous coronary intervention
PFO	patent foramen ovale
RCT	randomized controlled trial
RLM	restricted leaflet motion
SAPT	single antiplatelet therapy
SEV	self-expanding valve
SLT	subclinical leaflet thrombosis
TAVI	transcatheter aortic valve implantation
TEER	mitral transcatheter edge-to-edge repair
TIA	transient ischemic attack
TMVR	transcatheter mitral valve replacement
TR	tricuspid regurgitation
TTVR	transcatheter tricuspid valve replacement
UFH	unfractionated heparin
VKA	vitamin K antagonist
